# DNA barcoding reveals novel insights into pterygophagy and prey selection in distichodontid fishes (Characiformes: Distichodontidae)

**DOI:** 10.1002/ece3.1321

**Published:** 2014-11-19

**Authors:** Jairo Arroyave, Melanie L J Stiassny

**Affiliations:** Division of Vertebrate Zoology, Department of Ichthyology, American Museum of Natural HistoryCentral Park West at 79th St., New York, New York, 10024

**Keywords:** Ectoparasitic fin-eating behaviors, mtDNA, stomach contents, trophic ecology

## Abstract

DNA barcoding was used to investigate dietary habits and prey selection in members of the African-endemic family Distichodontidae noteworthy for displaying highly specialized ectoparasitic fin-eating behaviors (pterygophagy). Fin fragments recovered from the stomachs of representatives of three putatively pterygophagous distichodontid genera (*Phago*, *Eugnathichthys*, and *Ichthyborus*) were sequenced for the mitochondrial gene *co1*. DNA barcodes (*co1* sequences) were then used to identify prey items in order to determine whether pterygophagous distichodontids are opportunistic generalists or strict specialists with regard to prey selection and, whether as previously proposed, aggressive mimicry is used as a strategy for successful pterygophagy. Our findings do not support the hypothesis of aggressive mimicry suggesting instead that, despite the possession of highly specialized trophic anatomies, fin-eating distichodontids are opportunistic generalists, preying on fishes from a wide phylogenetic spectrum and to the extent of engaging in cannibalism. This study demonstrates how DNA barcoding can be used to shed light on evolutionary and ecological aspects of highly specialized ectoparasitic fin-eating behaviors by enabling the identification of prey species from small pieces of fins found in fish stomachs.

## Introduction

**Figure 1:**
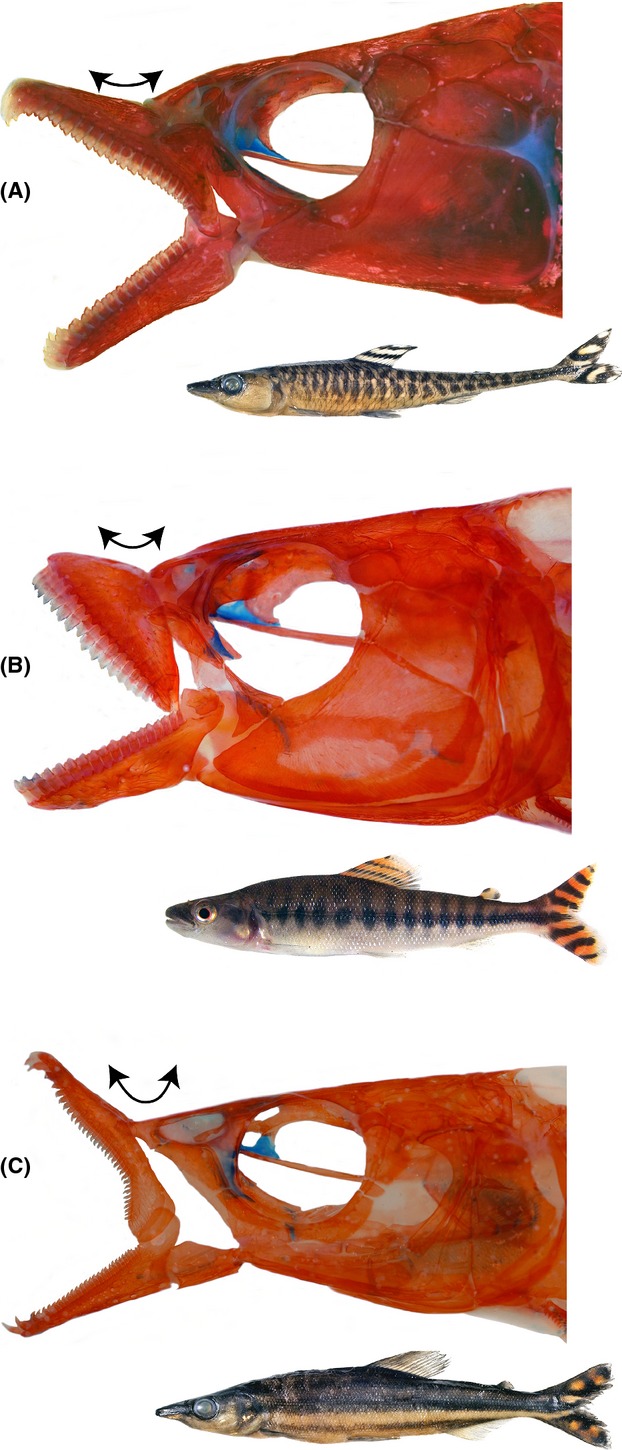
Variation in jaw anatomy in pterygophagous distichodontids represented in this study by the genera *Phago* (A), *Eugnathichthys* (B), and *Ichthyborus* (C).

Fishes of the family Distichodontidae, distributed throughout the freshwaters of much of sub-Saharan Africa and the Nile River basin, are one of the major groups of the African freshwater ichthyofauna (Vari 1979; Arroyave et al. 2013). Although moderate in diversity (∼100 spp. arrayed in 15 genera), distichodontids display remarkable variation in oral anatomy and exhibit a wide array of trophic ecologies, including detritivory, herbivory, insectivory, piscivory, and even ectoparasitic fin-eating behaviors (herein referred to as “pterygophagy”), facilitated by highly specialized jaw morphologies (Fig.1). Pterygophagy in distichodontid fishes, however, has not been investigated beyond the study that first documented this behavior more than 50 years ago (Matthes 1961) and two subsequent studies (Matthes 1964; Roberts 1990). Based on an observed similarity in caudal-fin coloration and patterning – as revealed by traditional stomach content analysis – between the ectoparasitic distichodontids *Eugnathichthys eetveldii* and *E*. *macroterolepis* and their putative prey *Synodontis decorus* and *Mesoborus crocodilus*, respectively, Roberts (1990) hypothesized that the barred caudal-fin pattern in pterygophagous distichodontids reflects a form of aggressive mimicry, allowing them to avoid detection by their monospecific prey. Four distichodontid genera – *Eugnathichthys*, *Belonophago*, *Ichthyborus*, and *Phago* – are reportedly ectoparasitic (i.e., feeding primarily on fish fins as adults) (Roberts 1990; Stiassny et al. 2013), but until the present study, there was virtually no information regarding the actual prey preferences of any of them.

Dietary information is critical for an understanding of community structure, ecological networks, and ecosystem functioning (Duffy et al. 2007), and can also inform conservation efforts for endangered species and/or threatened ecosystems (Marrero et al. 2004; Cristóbal-Azkarate and Arroyo-Rodríguez 2007). Approaches to determine the composition of animal diets include observation of foraging behavior, examination of stomach contents, and fecal analysis. Other methods such as fatty acid (FA) or stable isotope (SI) analyses, while capable of providing a substantive picture of energy and material flow through the food web, do not have the resolving power to accurately determine the relative contributions of different prey items to the diets of predators (Hardy et al. 2010). In stomach content and fecal analyses, food items are generally detected and identified either by direct visual inspection followed by traditional taxonomic identification or indirectly via DNA-based identification methods (e.g., DNA barcoding, DNA fingerprinting). The former approach, however, is often hampered by extensive prey digestion rendering only partial/incomplete prey items, frequently lacking species or even ordinal level diagnostic characteristics. Most DNA-based identification methods, on the other hand, allow for the identification and/or discrimination of prey items, often to the species level, even from partially digested tissue fragments. DNA barcoding, a molecule-based species identification method that uses short, standardized gene regions as species tags (e.g., the mitochondrial *co1* gene in animals, *rbcL* and *matK* chloroplast genes in land plants), offers an efficient and cost-effective alternative to determine the identity of prey items when they are not fully digested but can only be identified to a broad taxonomic rank (Valentini et al. 2009; Barnett et al. 2010), which is the case with fin fragments found in stomachs of pterygophagous distichodontid fishes (pers. obs.).

To further investigate pterygophagy in distichodontids and shed some light on evolutionary and ecological aspects of this highly unusual trophic strategy, DNA barcoding was used to identify prey species from fin fragments found in the stomachs of *Phago*, *Eugnathichthys*, and *Ichthyborus* specimens. Information on prey identity was then used to determine whether pterygophagous distichodontids are opportunistic generalists or strict specialists with regard to prey selection, and to test Roberts's (1990) hypothesis that aggressive mimicry is used as a strategy for successful pterygophagy in distichodontid fishes.

## Materials and Methods

### Specimen sampling and stomach content analysis

Fishes used in this study were collected and euthanized prior to preservation in accordance with recommended guidelines for the use of fishes in research (Nickum et al. 2004), and stress/suffering was ameliorated by minimizing handling and through the use of anesthetics prior to euthanasia. Because successful DNA extraction from formalin-fixed tissue remains challenging, if not unfeasible (Chakraborty et al. 2006), only specimens that were preserved in 95% EtOH were sampled for this study. A total of 43 ethanol-preserved individuals (14 *Phago*, seven *Eugnathichthys*, and 22 *Ichthyborus* specimens) were dissected for stomach contents analysis (Table1). Fin fragments found in stomachs were isolated, thoroughly cleaned, and rinsed with distilled water (to avoid contamination with predator-derived cells/tissues). Each was separately coded and kept in 95% EtOH. All dissected specimens, except for those corresponding to the species *Ichthyborus ornatus* (whose bodies are deposited in the teaching collection of the University of Kinshasa, Democratic Republic of Congo), are cataloged and stored in the ichthyology collection of the American Museum of Natural History (AMNH), available online at the museum's Vertebrate Zoology Collection Database (http://entheros.amnh.org/db/emuwebamnh/index.php).

**Table 1:** Specimens sampled for stomach contents analysis and their corresponding *co1* barcodes GenBank accession numbers

Genus	Species	Catalog #	Tissue #	GenBank Accession #
*Phago*	*P. boulengeri*	AMNH 259468	AMCC 215881	KP027369
		AMNH 259468	AMCC 215880	KP027370
		AMNH 259468	AMCC 215879	KP027371
		AMNH 259468	AMCC 215878	KP027372
		AMNH 259468	AMCC 215877	KP027373
		AMNH 259468	AMCC 215876	KP027374
		AMNH 259468	AMCC 215875	KP027375
		AMNH 259468	AMCC 215874	KP027376
		AMNH 259468	AMCC 215873	KP027377
		AMNH 259468	AMCC 215872	KP027378
		AMNH 259468	AMCC 215727	KP027379
		AMNH 260800	AMCC 216764	KP027380
	*P. intermedius*	AMNH 255629	AMCC 223226	KP027381
		AMNH 255148	AMCC 226195	KP027382
*Eugnathichthys*	*E. macroterolepis*	AMNH 263331	AMCC 227433	KP027383
		AMNH 263331	AMCC 227434	KP027384
		AMNH 263331	AMCC 227435	KP027385
		AMNH 263331	AMCC 227436	KP027386
		AMNH 263332	AMCC 227437	KP027387
		UKin^1^	n/a	KP027388
		UKin^1^	n/a	KP027389
*Ichthyborus*	*I. quadrilineatus*	AMNH 257060	AMCC 220511	KP027390
		AMNH 257060	AMCC 220512	KP027391
		AMNH 257060	t-113-11233	KP027392
	*I. ornatus*	UKin[Table-fn tf1-1]	T-0188	n/a
			T-0189	n/a
			T-0190	n/a
			T-0191	n/a
			T-0192	n/a
			T-0193	n/a
			T-0194	n/a
			T-0195	n/a
			T-0196	n/a
			T-0197	n/a
			T-0198	KP027393
			T-0199	n/a
			T-0200	KP027394
			T-0201	n/a
			T-0202	n/a
			T-0203	n/a
			T-0204	n/a
			T-0205	n/a
			T-0206	n/a

1 University of Kinshasa (teaching collection), uncataloged.

### DNA extraction, amplification, and sequencing

Total genomic DNA was extracted from both predator (i.e., pterygophagous distichodontids) and prey items (i.e., fin fragments found in their stomachs) using DNeasy Tissue Extraction Kit (Qiagen) following the manufacturer's protocol. DNA extracts were preserved in 95% EtOH and stored frozen. Amplification and sequencing of *co1* barcodes were carried out using Folmer et al.'s (1994) universal primers LCO1490 (5′-GGTCAACAAATCATAAAGATATTGG-3′) and HCO2198 (5′-TAAACTTCAGGGTGACCAAAAAATCA-3′). DNA amplification via polymerase chain reaction (PCR) was performed in a 25- *μ* L volume containing one Ready-To-Go PCR bead (GE Healthcare), 21  *μ* L of PCR-grade water, 1  *μ* L of each primer (10  *μ* mol/L), and 2  *μ* L of genomic DNA, under the following thermal profile: 5-min initial denaturation at 95°C, followed by 35 cycles of denaturation at 95°C for 60 s, annealing at 42°C for 60 s, and extension at 72°C for 90 s, followed by a 7-min final extension at 72°C. Double-stranded PCR products were purified using AMPure (Agencourt). Sequencing of each strand of amplified product was performed in a 5- *μ* L volume containing 1  *μ* L of primer (3.2  *μ* mol/L), 0.75  *μ* L of BigDye® Ready Reaction Mix, 1  *μ* L of BigDye® buffer, and 2.25  *μ* L of PCR-grade water. Sequencing reactions consisted of a 2-min initial denaturation at 95°C, followed by 35 cycles of denaturation at 95°C for 30 s, annealing at 45°C for 60 s, and extension at 72°C for 4 min, followed by a 3-min final extension at 72°C. All sequencing reactions were purified using CleanSEQ (Agencourt) and electrophoresed on an Applied Biosystems 3700 automated DNA sequencer in the AMNH Molecular Systematics Laboratories.

### Bioinformatics

Contig assemblage and sequence editing were performed using the software Geneious Pro v7.1.5 (Biomatters, available from http://www.geneious.com/). Species identification (of both predator and prey) was carried out using barcoding similarity methods based on the match between the query sequence and the reference sequences deposited in the Barcode of Life Database (BOLD) and GenBank using NCBI BLAST (Altschul et al. 1990; Johnson et al. 2008). The best match (“top hit”) was taken as the best estimate of taxonomic identity, with matches ≥98% similar assumed to be conspecifics, thus allowing an admittedly arbitrary, but operational threshold of a 2% difference between query and reference sequences to account for intraspecific variation (Jarman et al. 2004). In those cases where the best estimate of taxonomic identity was ambiguous (i.e., >2% *co1* divergence), available specimens of potential prey species (i.e., species living in sympatry with the sampled pterygophagous distichodontids) previously unrepresented in GenBank/BOLD databases (Table2) were sequenced for *co1* with the goal of confirming prey identity to the species level.

**Table 2:** Available specimens of potential prey species (i.e., species living in sympatry with the sampled pterygophagous distichodontids) previously unrepresented in GenBank/BOLD databases and sequenced for *co1* with the goal of confirming prey identity to the species/subspecies level

Species	Catalog #	Tissue #	GenBank Accession #
*Chrysichthys nigrodigitatus*	AMNH 263329	AMCC 227431	KP027395
*Chrysichthys ornatus*	AMNH 260757	AMCC 215865	KP027396
*Oreochromis lepidurus*	AMNH 263330	AMCC 227432	KP027397
*Sarotherodon galilaeus boulengeri*	AMNH 260750	AMCC 215857	KP027398
*Tylochromis lateralis*	AMNH 241101	t-031-3016	KP027399

## Results

Overall, 55 fin fragments were recovered from the stomachs of 23 of the 43 sampled specimens, and as expected it was not possible to visually discern prey species from fin remains. With the exception of all 19 *Ichthyborus ornatus* specimens (which had whole fish, but no fin fragments in their stomachs) and an individual of *Eugnathichthys macroterolepis* (which had stomach contents later identified via *co1* barcoding as horn snails), all remaining stomachs contained between one and five distinct fin fragments.

DNA barcodes confirmed the species identity of all individuals of the pterygophagous distichodontid species investigated in this study (i.e., *Phago boulengeri*, *P. intermedius*, *Eugnathichthys macroterolepis*, *Ichthyborus quadrilineatus*, and *I. ornatus*). Amplification and/or sequencing of *co1* failed in 10 of the 55 fin fragments. The results of the BLAST search for each of the 45 successfully sequenced fin fragments are presented in Table3. The *co1* barcodes from a total of 19 fish species in nine families and four orders were identified as being identical or fairly similar to those from the fin fragments found in the examined stomachs, with most barcode matches being >99% similar. Barcodes from fin fragments found in a single *Ichthyborus* and four *Phago* specimens BLASTed to conspecifics (i.e., *I*. *quadrilineatus* and *P*. *boulengeri*, respectively), suggesting a not infrequent occurrence of cannibalism among some pterygophagous lineages.

**Table 3:** Results of the BLAST search for each of the 45 successfully sequenced fin fragments retrieved from the stomachs of the pterygophagous distichodontid species sampled in this study

Genus	Species	Catalog #	Fin Fragment ID	Best Match (“Top Hit”)	Family, Order	% Similarity
*Phago*	*P. boulengeri*	AMNH 259468	215881-a	*Brycinus imberi*	Alestidae, Characiformes	100
		AMNH 259468	215879-a	*Sarotherodon galilaeus* 1	Cichlidae, Perciformes	99.4
			215879-b	*Sarotherodon galilaeus* 1	Cichlidae, Perciformes	99.4
			215879-c	*Hemichromis bimaculatus*	Cichlidae, Perciformes	94.3
			215879-e	*Sarotherodon galilaeus* 1	Cichlidae, Perciformes	99.4
		AMNH 259468	215878-a	*Synodontis contracta*	Mochokidae, Siluriformes	98.6
			215878-b	*Synodontis nigriventris*	Mochokidae, Siluriformes	98.0
			215878-c	*Synodontis nigriventris*	Mochokidae, Siluriformes	98.0
		AMNH 259468	215877-a	*Phago boulengeri*	Distichodontidae, Characiformes	100
			215877-b	*Sarotherodon galilaeus* 1	Cichlidae, Perciformes	99.2
			215877-c	*Sarotherodon galilaeus* 1	Cichlidae, Perciformes	99.2
		AMNH 259468	215876-b	*Tylochromis polylepis* 2	Cichlidae, Perciformes	97.5
			215876-c	*Sarotherodon galilaeus* 1	Cichlidae, Perciformes	99.4
			215876-d	*Tylochromis polylepis* 2	Cichlidae, Perciformes	97.1
		AMNH 259468	215875-a	*Phago boulengeri*	Distichodontidae, Characiformes	100
			215875-c	*Sarotherodon galilaeus* 1	Cichlidae, Perciformes	99.2
		AMNH 259468	215874-a	*Sarotherodon galilaeus* 1	Cichlidae, Perciformes	99.4
			215874-b	*Sarotherodon galilaeus* 1	Cichlidae, Perciformes	99.4
		AMNH 259468	215873-a	*Synodontis nigriventris*	Mochokidae, Siluriformes	98.0
			215873-b	*Phago boulengeri*	Distichodontidae, Characiformes	100
			215873-c	*Brycinus comptus*	Alestidae, Characiformes	100
			215873-d	*Brycinus comptus*	Alestidae, Characiformes	100
		AMNH 259468	215872-a	*Phago boulengeri*	Distichodontidae, Characiformes	100
			215872-b	*Synodontis nigriventris*	Mochokidae, Siluriformes	98.7
		AMNH 259468	215727-a	*Chrysichthys nigrodigitatus*	Claroteidae, Siluriformes	92.5
			215727-b	*Chrysichthys nigrodigitatus*	Claroteidae, Siluriformes	92.5
		AMNH 260800	216764-a	*Heterotis niloticus*	Arapaimidae, Osteoglossiformes	100
			216764-b	*Heterotis niloticus*	Arapaimidae, Osteoglossiformes	100
			216764-c	*Heterotis niloticus*	Arapaimidae, Osteoglossiformes	100
	*P. intermedius*	AMNH 255629	223226-a	*Alestopetersius* sp. “mbuji”	Alestidae, Characiformes	99.2
			223226-b	*Alestopetersius* sp. ‘mbuji”	Alestidae, Characiformes	99.2
*Eugnathichthys*	*E. macroterolepis*	AMNH 263331	227433-a	*Chrysichthys ornatus* 3	Claroteidae, Siluriformes	97.7
		AMNH 263331	227435-a	*Chrysichthys ornatus* 3	Claroteidae, Siluriformes	97.1
		AMNH 263331	227436-a	*Awaous ocellaris*	Gobiidae, Perciformes	88.6
		AMNH 263332	227437-a	*Trachinotus goreensis*	Carangidae, Perciformes	100
			227437-b	*Chrysichthys auratus* 4	Claroteidae, Siluriformes	96.7
		UKin uncat.	UK-1-a	*Trachinotus goreensis*	Carangidae, Perciformes	100
			UK-1-b	*Oreochromis mossambicus* 5	Cichlidae, Perciformes	96.9
		UKin uncat.	UK-2-a	*Chrysichthys auratus* 4	Claroteidae, Siluriformes	96.7
			UK-2-b	*Chrysichthys ornatus* 3	Claroteidae, Siluriformes	96.9
*Ichthyborus*	*I. quadrilineatus*	AMNH 257060	220511-a	*Chrysichthys auratus*	Claroteidae, Siluriformes	93.3
			220511-b	*Chrysichthys auratus*	Claroteidae, Siluriformes	93.3
			220512-c	*Synodontis annectens*	Mochokidae, Siluriformes	99.6
			220512-d	*Ichthyborus quadrilineatus*	Distichodontidae, Characiformes	99.7
			113-11233-a	*Hepsetus odoe*	Hepsetidae, Characiformes	88.5

1 Confirmed as subspecies *Sarotherodon galilaeus boulengeri* (>99.7% *co1* similarity).

2 Confirmed as *Tylochromis lateralis* (99.3% *co1* similarity).

3 Confirmed as *Chrysichthys ornatus* (>99.2% *co1* similarity).

4 Confirmed as *Chrysichthys nigrodigitatus* (99.7% *co1* similarity).

5 Confirmed as *Oreochromis lepidurus* (99.9% *co1* similarity).

The BLASTing of *co1* barcodes from 15 of the 45 fin fragments resulted in best matches (“top hit”) that were <98% similar, and therefore, whose best estimate of taxonomic identity could only be made above the species level. Although some prey species were not represented in either the BOLD or the GenBank databases, in all cases match percentages to query sequences were still sufficient to at least confidently assign prey items to genus (or family in the case of the horn snails recovered from one *Eugnathichthys* specimen). In eight of the 15 instances of questionable identification, species identity was later confirmed using *co1* barcodes generated in this study from potential prey species collected in sympatry with the sampled pterygophages. Likewise, all prey items initially identified as *Sarotherodon galilaeus* were confirmed as subspecies *S. galilaeus boulengeri* using *co1* barcodes previously unrepresented in databases (Table3).

## Discussion

Pterygophagous distichodontids – represented in this study by members of the genera *Phago*, *Eugnathichthys*, and *Ichthyborus* – prey on fishes from a wide phylogenetic spectrum that includes at least nine teleostean families (Arapaimidae, Alestidae, Distichodontidae, Hepsetidae, Claroteidae, Mochokidae, Carangidae, Gobiidae, and Cichlidae) from four orders (Osteoglossiformes, Characiformes, Siluriformes, and Perciformes). These findings suggest that the ecological strategy involved in distichodontid pterygophagy is one of prey generalization rather than specialization (*contra* Roberts (1990)). Interestingly, in these fishes, a notably high degree of morphological and behavioral specialization underpins a highly specialized feeding modality, which in turn facilitates the utilization of a wide spectrum of potential prey. Although the trade-offs between specialization and generalization are complex and multifactorial (Hawkins 1994; Thompson 1994), ecological models have shown that the more polyphagous the predator, the less vulnerable it is to scarcity and/or extinction of a particular prey species (Montoya et al. 2006). The present finding that *Phago boulengeri* from the Congo River basin feeds on the fins of *Heterotis niloticus*, a species native to the Sahelo-Sudanese region (Daget 1984), and only recently (year 1960) introduced into the Congo basin (FAO 2005), further reinforces the idea that pterygophagy in distichodontids facilitates opportunistic feeding on a wide range of available prey regardless of historical context.

The findings of this study further indicate that adult *Eugnathichthys macroterolepis*, although primarily pterygophagous can, on occasion, exploit alternative food resources. The stomach of one individual collected near the mouth of the Congo River contained numerous mollusks identified as horn snails (family Potamididae) via DNA barcoding. Interestingly, these snails were intact but devoid of shells implying that *E. macroterolepis* used its strong jaws (Fig.1B) to grasp the exposed foot of each snail to twist it out of its shell before consumption, presumably in a manner analogous to that of the Lake Victorian “snail shelling” cichlids (Greenwood 1973). Similarly, our results indicate that at least one species of *Ichthyborus*, *I. ornatus*, is not an obligate pterygophage, as all 19 specimens examined here had intact, or partially digested, fishes distending their stomachs. *Belonophago* is the only pterygophagous distichodontid genus not included in the current study due to lack of available ethanol-preserved material. However, observation of aquarium-held specimens of *Belonophago tinanti* indicates that it is an obligate pterygophage feeding exclusively on caudal fins from a wide range of species, although prey preferences in wild populations remain to be determined.

**Figure 2:**
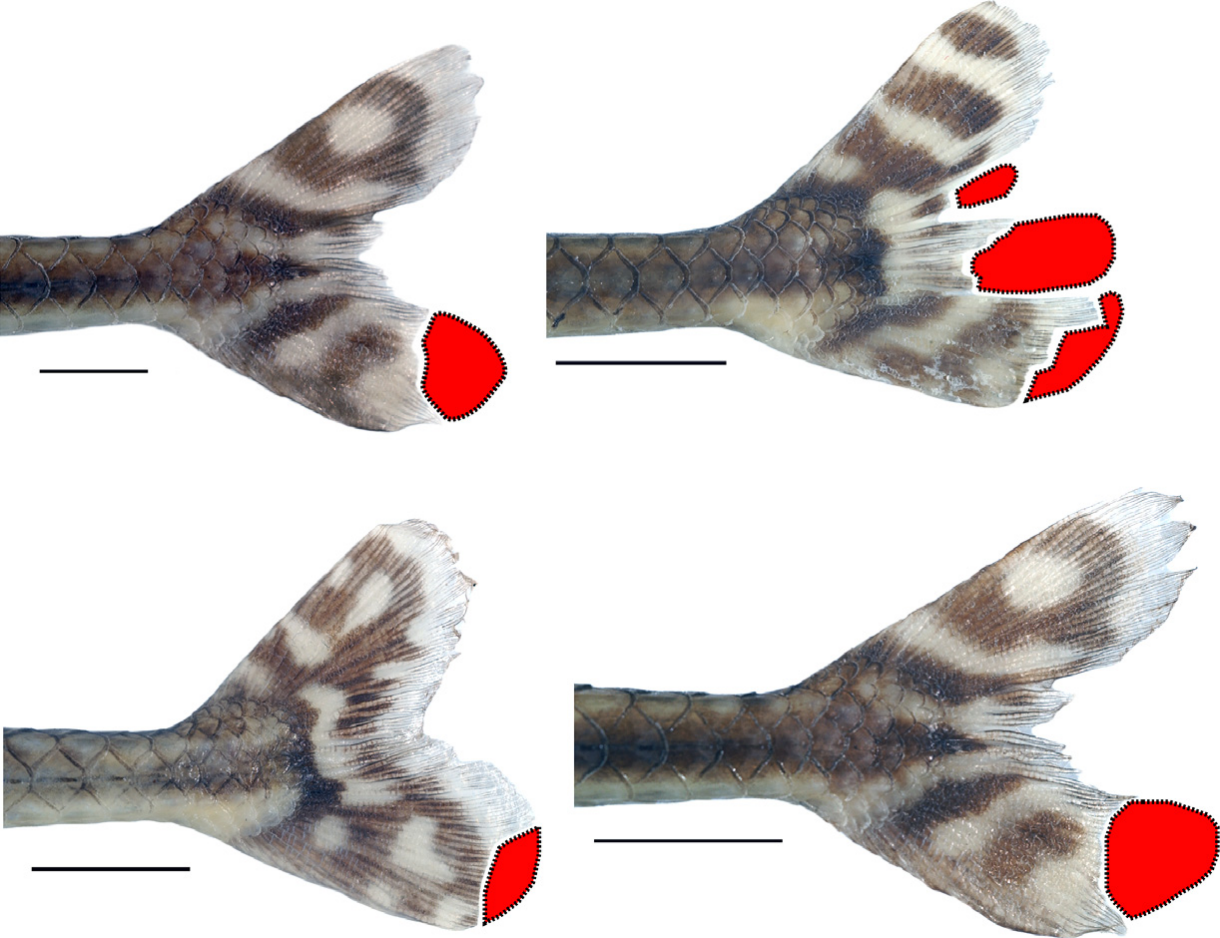
Characteristically damaged fins in *Phago* specimens victims of pterygophagy. Scale bars represent 1 cm.

Our results indicate that at least two species of pterygophagous distichodontids (i.e., *Phago boulengeri* and *Ichthyborus quadrilineatus*) engage in cannibalism. This unanticipated finding underscores the manifestly opportunistic prey selection strategy of fin-eating distichodontids, allowing them to feed on any accessible resources, even members of their own species. We note in this regard that examination of the caudal fins of over 70 preserved specimens of *P. boulengeri* held in the AMNH collection reveals a high proportion (>20%) of fins showing clear evidence of attack. The damaged fins characteristically are missing a discrete block of fin rays that appear to have been cleanly sheared off (Fig.2). While it is not possible to ascertain whether all of these *Phago* specimens were subject to intraspecific attack, or attack by other sympatric pterygophagous distichodontids, such a high incidence of fin damage in the species is noteworthy. Although cannibalism in fishes is widespread and has been documented in numerous families from across the teleost tree of life (Smith and Reay 1991), most known instances represent filial cannibalism, in which adults consume all or part of their own offspring (Manica 2002). The present study appears to be the first to report the occurrence of ectoparasitic cannibalism by pterygophagous fishes.

**Figure 3:**
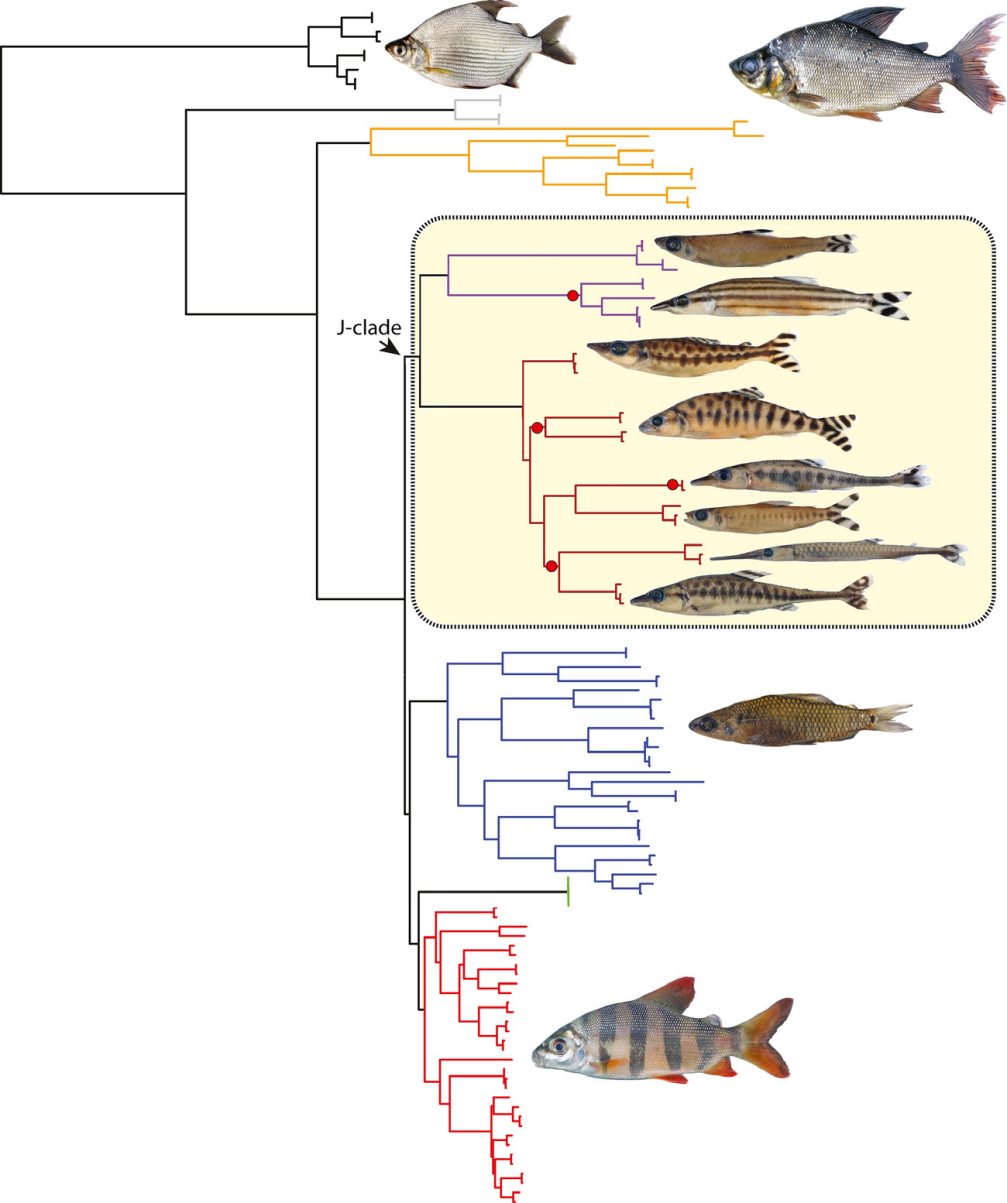
Citharinoid phylogeny (modified after Arroyave et al. 2013), with the distichodontid “J clade” highlighted and pterygophagous lineages indicated by red circles.

In an early study investigating fin-eating behavior in distichodontid fishes, Roberts (1990) proposed that aggressive mimicry is used as a strategy for successful pterygophagy in *Eugnathichthys*. While aggressive mimicry appears to be the preferred strategy in the few lepidophagous and pterygophagous freshwater fishes so far investigated (Hori and Watanabe 2000; Sazima 2002), in the case of the distichodontids investigated here our results do not support that hypothesis. The striking-barred coloration and patterning of the caudal fins of *Eugnathichthys eetveldii* and *E. macroterolepis* first noted by Roberts (1990) is recognized here as a character diagnostic of a clade of distichodontid fishes (designated the “J clade” by Arroyave et al. (2013), p. 11, fig. 4), and no other distichodontids share this feature (Fig.3). While the “J clade” does include all pterygophagous genera, it also includes three genera with members that are either piscivores (*Mesoborus*) or insectivores (*Hemistichodus* and *Microstomatichthyoborus*). The topology of Arroyave et al.'s (2013) distichodontid tree (Fig.3) suggests that this caudal patterning is likely an exaptation (*sensu* Gould and Vrba (1982)) rather than an adaptation for aggressive mimicry. The results of this study therefore suggest that Roberts's (1990) findings (i.e., similar caudal coloration between predator and prey) are simply coincidental. The fact that none of the prey species identified in the present study (with the exception of the cannibalized individuals) display a caudal-barring pattern or coloration similar to that found in their pterygophagous predators further refutes the notion that fin-eating distichodontids are utilizing aggressive mimicry as a strategy for successful pterygophagy.

Although highly unusual, pterygophagy in teleost fishes is not exclusive to distichodontids and has been documented in a few other groups, such as piranhas of the genus *Serrasalmus* (Northcote et al. 1986, 1987; Nico and Taphorn 1988), blennies of the genus *Aspidonotus* (Eibl-Eibesfeldt 1959; Randall and Randall 1960; Kuwamura 1983), and cichlids of the genera *Docimodus* (Ribbink 1984) and *Genyochromis* (Ribbink et al. 1983). Nevertheless, information on predator-prey interactions for most of these is virtually nonexistent, and the present study represents the first assessment of prey preferences in a group of highly specialized pterygophagous fishes. Although dietary studies such as the one presented here are primarily qualitative, basic knowledge of species-level interactions between predators and prey constitutes the very first step in determining more precise food-web characterizations in complex tropical freshwater ecosystems.
